# Pharmacologic Treatment of Pulmonary Hypertension Due to Heart Failure with Preserved Ejection Fraction: Are There More Arrows on Our Bow?

**DOI:** 10.3390/jcm13226867

**Published:** 2024-11-14

**Authors:** Daniele Masarone, Fabio Valente, Marina Verrengia, Carla Contaldi, Vito di Palma, Luigi Falco, Dario Catapano, Emilio di Lorenzo

**Affiliations:** Department of Cardiology, AORN dei Colli-Monaldi Hospital, Via Leonardo Bianchi 1, 80131 Naples, Italy

**Keywords:** pulmonary hypertension, heart failure with preserved ejection fraction, levosimendan

## Abstract

Pulmonary hypertension (PH) associated with heart failure with preserved ejection fraction (PH-HFpEF) represents a frequent form of PH related to left ventricular dysfunction. The pathophysiology of PH-HFpEF is intricate, and varied and includes vascular, cardiac, and pulmonary factors that contribute synergistically to developing this clinical syndrome. Improved knowledge of the pathophysiology of PH-HFpEF has paved the way for the use of new drugs such as angiotensin receptor neprilysin inhibitors (ARNIs), non-steroidal mineral corticoid receptor antagonist (nsMRA), sodium-glucose cotransporter inhibitors (SGLT2is), levosimendan, and glucagon-like peptide 1 (GLP-1) agonists. ARNIs are a widely used drug for the treatment of PH associated with heart failure with reduced ejection fraction. They have also recently been used in PH-HFpEF patients with hemodynamic benefits that need to be confirmed in future research. Finerenone is an innovative non-steroidal mineralocorticoid receptor antagonist that exhibits notable cardioprotective and renoprotective properties in individuals suffering from chronic diabetic kidney disease. It also enhances outcomes for patients with heart failure, whether they have mildly reduced or preserved ejection fraction. Moreover, in experimental studies, finerenone has been found to lower pulmonary artery pressure, reduce muscularization, and decrease the wall thickness of pulmonary arteries. SGLT2i have revolutionized the treatment of patients with heart failure irrespective of left ventricular ejection fraction, and their treatment is also associated with an improvement in the hemodynamics profile in patients with PH-HFpEF. Levosimendan is a widely used inodilator in the treatment of acute and advanced heart failure. In addition, its use in patients with PH-HFpEF (supported by the positive effects on pulmonary hemodynamics that levosimendan exerts) has recently demonstrated hemodynamic benefit in a small phase 2 study that paved the way for phase 3 studies and the creation of an oral formulation of levosimendan. Finally, GLP1 agonists are a class of drugs that, in preliminary evidence, have shown a positive effect on cardiac hemodynamics, mainly by facilitating left ventricular unloading. These effects, along with the reduction in insulin resistance and weight loss, likely lead to beneficial outcomes for PH-HFpEF patients, especially those with obesity as a comorbidity.

## 1. Introduction

According to the European Society of Cardiology guidelines, pulmonary hypertension (PH) is a chronic, progressive condition characterized by a pulmonary arterial mean pressure (PAMP) exceeding 20 mmHg [1]. It can lead to right ventricular failure and cardiovascular death [2]. The most prevalent type of PH arises from left ventricular heart disease (group 2 PH) [3], particularly affecting patients with heart failure (HF), especially those with heart failure with preserved ejection fraction (HFpEF) [4]. A pulmonary artery systolic pressure (PASP) above 35 mmHg is frequently observed in HFpEF and serves as a diagnostic criterion in two established scoring systems for the early detection of patients with HFpEF: the H2FPEF score and the HFA-PEFF scoring algorithm [5,6]. However, PH associated with HFpEF (PH-HFpEF) is related to more advanced disease [7] and correlates with more severe symptoms [8], along with increased morbidity and mortality rates [9]. Recent breakthroughs have greatly improved our understanding of the pathophysiology of PH-HFpEF, paving the way for new treatments such as angiotensin receptor neprilysin inhibitors (ARNIs), non-steroidal mineral corticoid receptor antagonist (nsMRA), sodium-glucose cotransporter inhibitors (SGLT2is), levosimendan and glucagon-like peptide 1 (GLP-1) agonists. This paper intends to summarize the current evidence on the clinical applications of these innovative pharmacological options and discuss their potential future roles in this intricate clinical scenario.

## 2. Epidemiology of PH-HFpEF

The prevalence of PH-HFpEF remains uncertain and varies significantly based on the type of study (randomized clinical trials versus cohort studies), the diagnostic method employed for detecting PH-HFpEF (standard echocardiography versus right heart catheterization), and the specific tricuspid regurgitant velocity and/or PAMP thresholds applied for diagnosis [10]. In the TOPCAT (Spironolactone for Heart Failure with Preserved Ejection Fraction) trial, 36% of patients had PH, identified by a tricuspid regurgitant velocity greater than 2.9 m/s [11]. The PARAGON-HF (Prospective Comparison of ARNI With ARB Global Outcomes in HF With Preserved Ejection Fraction) study yielded similar findings, indicating that 31% of patients had PH-HFpEF, also defined by a tricuspid regurgitant velocity exceeding 2.9 m/s [12]. In a community-based study involving 244 patients with HFpEF (the Olmsted study), the incidence of PH-HFpEF, defined as a systolic pulmonary arterial pressure (SPAP) greater than 35 mmHg, was found to be 83% [13]. A more recent retrospective study analyzing 464,438 acute HFpEF hospitalizations reported a prevalence of PH at 27.1% [14]. While the various clinical studies demonstrate differing prevalence rates, they consistently reveal one crucial finding: increased pulmonary arterial pressure is directly linked to clinical outcomes. A multicenter study that included 388 HF patients, among them 96 with HFpEF, found that for every five mmHg increase in SPAP, there was a 9% rise in mortality (*p* < 0.0008) [15]. Furthermore, Lam et al. indicated that each 10% rise in SPAP was associated with a 28% increased risk of cardiovascular events over a 3-year follow-up period [13].

## 3. Pathophysiology of PH-HFpEF

The pathophysiology of PH-HFpEF is complex and varied, and it is still not fully elucidated today [16]. As illustrated in Table 1, multiple factors have classically been associated with PH in HFpEF; however, newer pathophysiological factors have more recently been identified that have allowed the testing of new drugs with specific mechanisms of action.

### 3.1. Hemodynamic of PH-HFpEF

The key feature of HFpEF’s pathophysiology is the left ventricle’s impaired relaxation and stiff myocardium, resulting in abnormal filling pressure increases during volume overload [17]. These elevated left ventricular filling pressures are transferred passively to the left atrium, leading to a rise in left atrial pressure and to the pulmonary veins, which results in increased PAMP. This scenario culminates in isolated post-capillary PH. High hydrostatic pressure causes ‘lung-capillary stress failure due to barotrauma’, characterized by endothelial dysfunction, which elevates the permeability of alveolar units, reduces nitric oxide production, and increases endothelin levels [18]. Ongoing increases in PAMP lead to structural changes in small pulmonary venules, including luminal narrowing, more significant neointimal thickening, and medial hypertrophy. Additionally, arterioles and capillaries experience smooth muscle cell hypertrophy, proliferation, and intimal fibrosis. These vascular changes raise pulmonary vascular resistance, forming the pathophysiological foundation for combined pre-capillary and post-capillary pulmonary hypertension [19].

### 3.2. Neurohormonal Factors and HFpEF

An imbalance in the natriuretic peptide system could directly affect the development of PH-HFpEF. For instance, lower levels of circulating natriuretic peptides are commonly seen in patients with obesity and insulin resistance, both of which are independently linked to PH [20]. Additionally, activating A- and B-type natriuretic peptide receptors enhances metabolic fat utilization by increasing intracellular cGMP, thereby reducing obesity and insulin resistance risks [21].

The natriuretic peptide type C also has positive effects in preventing the development of PH-HFpEF; in fact, an agonist of the natriuretic peptide receptor type C, NPR-C, has recently been shown to reduce right ventricular systolic pressure and PASP in an experimental mice model [22]. Furthermore, it is interesting to note that the vasodilator effect of NPR-C signaling is increased in the presence of inhibition of nitric oxide synthase [23].

This is particularly important in PH-HFpEF patients, in which endothelial dysfunction and, thus, a reduction or loss of the nitric oxide pathway are pathophysiologic hallmarks.

The natriuretic peptide system also protects myocardial structure [24]. In animal models, stimulating A-type natriuretic peptide receptors has been found to decrease interstitial fibrosis and cardiomyocyte hypertrophy. Furthermore, natriuretic peptides lower the production of endothelin-1 (an isopeptide capable of producing intense and prolonged vasoconstriction of the pulmonary arteries and veins, as well as a mitogenic effect on pulmonary vascular smooth muscle cells and the induction of matrix production by the vascular wall), while boosting nitric oxide release [25].

Considering this pathophysiological evidence, it can be inferred that a relative deficiency of natriuretic peptides may directly or indirectly induce the hemodynamic and structural changes associated with PH-HFpEF [26]. Thus, utilizing drugs that increase circulating levels of natriuretic peptides might be beneficial in this clinical context.

### 3.3. Metabolic Factors and HFpEF

Not every patient with HFpEF develops PH, suggesting that elevated left ventricular filling pressures and structural changes in the pulmonary capillaries and arterioles are not the only factors causing PH-HFpEF [27]. Recent studies indicate that conditions such as obesity, diabetes, hyperglycemia, insulin resistance, and hypertension might contribute to the development of PH-HFpEF [28,29]. In a widely utilized leptin-deficient mouse model, which simulates obesity and diabetes, indications of macrophage infiltration, myofibroblast proliferation, and remodeling of the pulmonary artery were detected as early as 12 weeks [30,31]. In a distinct mouse model subjected to a high-fat diet—intended to replicate insulin resistance and type 2 diabetes—pulmonary artery remodeling, elevated pulmonary artery pressure, and right ventricular hypertrophy were observed [32]. These alterations were particularly significant in certain inbred and wild-derived mouse strains, such as the AKR/J strain, which is especially susceptible to developing high-fat diet-induced PH-HFpEF.

In contrast, the NOD/ShiLtJ mouse strain, representing autoimmune type 1 diabetes, exhibited resistance to HFD-induced PH-HFpEF. On the other hand, the NON/ShiLtJ mouse strain, which is vulnerable to HFD-induced type 2 diabetes, showed susceptibility to developing PH-HFpEF. These results suggest a substantial role of type 2 diabetes in the pathophysiology of PH-HFpEF [33]. In the ZSF1 experimental mouse model—which mimics essential aspects of metabolic syndrome, including obesity, hyperglycemia, and hypertension—increased left ventricular filling pressures and pulmonary arterial hypertension were detected as early as 20 weeks of age [34,35].

This model also highlighted a correlation between reduced glucose uptake by skeletal muscle and elevated systolic pulmonary pressure [36], indicating that the pathophysiology of PH-HFpEF involves several intricate mechanisms.

Additionally, research on a rat model indicated that rats given a high-fat diet and olanzapine (which simulates metabolic syndrome) exhibited heightened pulmonary vascular resistance and remodeling compared to sham rats [37].

Finally, various large animal models, like older dogs with diastolic dysfunction and Ossabaw pigs with metabolic syndrome, have shown that aging plays a role in developing PH-HFpEF [38].

## 4. Evidence Supporting the Use of ARNI and nsMRA in PH-HFpEF

In recent years, numerous studies have indicated that ARNIs enhance right ventricular function and alleviate pulmonary hypertension in patients with heart failure and reduced ejection fraction [39], even during later stages of the disease [40]. Furthermore, recent research has highlighted the potential utility of the use of ARNIs also in patients with PH-HFpEF. A retrospective study involving 18 patients with PH-HFpEF demonstrated that treatment with sacubitril valsartan (at a dose of 24/26 mg twice daily) led to a mean follow-up period of 99 days in a statistically significant decrease in PAMP from 33 to 27 mmHg (*p* < 0.05) and in pulmonary capillary wedge pressure (PCWP) from 22 to 16 mmHg (*p* < 0.05) as measured by right cardiac catheterization. Importantly, this study found no statistically significant changes in echocardiographic parameters related to left ventricular systolic and diastolic function, as well as cardiac output and cardiac index (assessed via right heart catheterization). These findings suggest that the observed benefits primarily stem from sacubitril/valsartan’s vasodilatory effects within the pulmonary vascular system rather than improvements in left ventricular function [41]. More recently, a single-arm study including 14 consecutive outpatients with symptomatic PH-HFpEF who were subjected to CardioMEMS implantation found that treatment with sacubitril/valsartan led to a significant reduction in PAMP by 4.99 mmHg (95% CI −5.55 to −4.43). Notably, an increase in PAMP of +2.84 mmHg (95% CI; +2.26 to +3.42) was observed following the withdrawal of sacubitril/valsartan therapy [42]. Although this evidence remains limited, the underlying pathophysiological mechanisms and initial clinical findings suggest that ARNIs could be a therapeutic option for patients with PH-HFpEF [43]. While not specified in the European Society of Cardiology’s guidelines for treating pulmonary arterial hypertension and approved solely in the United States for HFpEF, the authors advocate for the use of sacubitril/valsartan in patients with PH-HFpEF, especially those with a left ventricular ejection fraction near 50%. This recommendation stems from a sub-analysis of the PARAGON-HF study (Angiotensin Receptor Neprilysin Inhibition in Heart Failure with Preserved Ejection Fraction), indicating that ARNI treatment benefits specific groups, such as women and patients with a left ventricular ejection fraction ranging from 45% to 57% [44].

Finerenone is a newly developed nsMRA with a strong binding affinity, high selectivity for the mineralocorticoid receptor, and a shorter plasma half-life than traditional steroid mineralocorticoid receptor antagonists [45]. In the Effect of Finerenone on Chronic Kidney Disease Outcomes in Type 2 Diabetes (FIDELIO-DKD) and in the Cardiovascular Events with Finerenone in Kidney Disease and Type 2 Diabetes (FIGARO-DKD) trials, finerenone demonstrated a significant protective effect on both the heart and kidneys, in patients with diabetic chronic kidney disease [46,47]. Additionally, the FINEARTS-HF study showed that finerenone considerably reduced the incidence of worsening heart failure events and cardiovascular-related deaths compared to placebo in patients with heart failure and mildly reduced or preserved ejection fraction [48].

Current findings indicate that finerenone benefits patients with PH-HFpEF based on experimental data. In a rat model of monocrotaline and sugen/hypoxia, finerenone leads to a reduction in PAMP and decreases in muscularization and wall thickness of pulmonary arteries [49]. Although evidence remains sparse, the authors concur that future studies and specific trials will validate eplerenone’s beneficial hemodynamic effects in patients with PH-HFpEF.

## 5. Evidence Supporting the Use of SGLT2is and Levosimendan in PH-HFpEF

**Figure 1:**
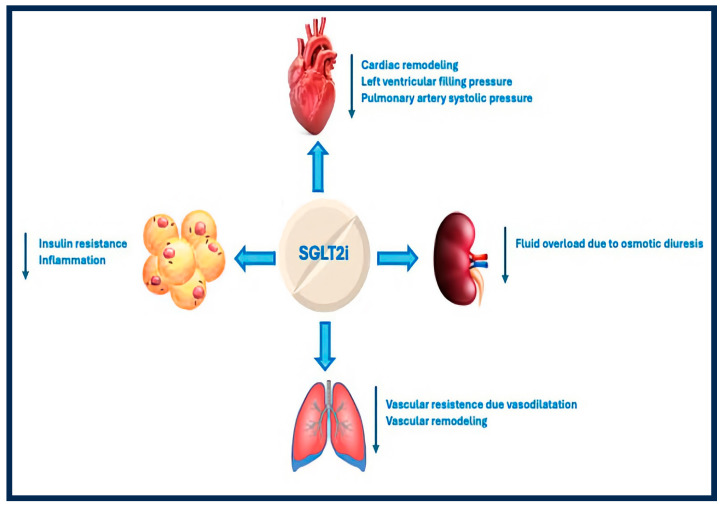
Beneficial effects of SGLT2i in patients with PH-HFpEF.

SGLT2is exhibits several effects that can benefit patients with PH-HFpEF (Figure 1). They reduce insulin resistance and enhance myocardial substrate utilization by promoting fatty acid oxidation and lowering glycolysis [50]. Additionally, these drugs lead to glycosuria, which causes osmotic diuresis, thereby decreasing PCWP and PASP [51]. Moreover, SGLT2is protects kidney function by enhancing diuresis and optimizing intraglomerular hemodynamics, which may further support their positive impact on PH-HFpEF. Notably, fluid overload and endothelial dysfunction, common in chronic kidney disease patients, are linked to a heightened risk of PH [52]. In addition, SGLT2is reduces inflammation and cardiac remodeling, preventing PH-HFpEF. In diabetic rat studies, blocking sodium/glucose cotransporter 2 via either a non-specific inhibitor (phlorizin) or a targeted one (canagliflozin) demonstrated a dose-dependent direct vasodilation of pulmonary arteries, with canagliflozin showing a more pronounced effect (60% vs. 40% relaxation) [53]. Recent research using a monocrotaline-induced pulmonary arterial hypertension rat model showed that empagliflozin treatment lowered pulmonary vascular resistance significantly (65.58 ± 6.02 vs. 43.8 ± 4.4 mmHg; *p* < 0.01) and decreased PAMP values (26.72 ± 5.8 vs. 21.25 ± 4.4 mmHg; *p* < 0.05), along with reducing pulmonary artery medial wall thickness (50.8 ± 2.2 vs. 44.7 ± 1.1 mm; *p* < 0.01) [54]. Initial clinical evidence emphasizes the beneficial effects of SGLT2 on patients with PH-HFpEF. In a Japanese trial with 78 HFpEF patients, dapagliflozin (5 mg as additional treatment) notably lowered the occurrence of exercise-induced pulmonary hypertension (PASP > 50 mmHg) when compared to the placebo group (*p* = 0.008) [55]. Additionally, the trial Empagliflozin Effects on Pulmonary Artery Pressure in Patients with Heart Failure (EMBRACE-HF) examined the impact of empagliflozin (10 mg) on PAMP levels in patients with HFmrEF/HFpEF [56]. The follow-up findings indicated that patients receiving empagliflozin saw a notable decrease in diastolic pulmonary pressure of 1.5 mmHg at eight weeks (95% CI, 0.2–2.8; *p* = 0.02) and 1.7 mmHg at 12 weeks (95% CI, 0.3–3.2; *p* = 0.02) as measured by CardioMEMS, in comparison to the placebo group. Similarly, the Cardiac and Metabolic Effects of Dapagliflozin in Heart Failure with Preserved Ejection Fraction (CAMEO-DAPA) trial assessed dapagliflozin (10 mg) in 38 HFpEF patients, showing significant decreases in PCWP (33.5 vs. 27 mmHg; *p* = 0.02), right atrial pressure (19.9 vs. 16.5 mmHg; *p* = 0.01), and PAMP (49.9 vs. 44.7 mmHg; *p* = 0.02) [57]. Despite the limited sample sizes, the results of these studies indicate that SGLT2is may effectively enhance pulmonary hemodynamics and address vascular issues in PH-HFpEF patients.

We strongly believe that the different mechanisms through which SGLT2is acts make them the treatment of choice for patients with PH-HFpEF. Therefore, according to international guidelines, they must be used in all with HFpEF [58,59], especially those showing direct echocardiographic signs of PH, like elevated pulmonary systolic pressure, or indirect signs, such as a ventricular D shape, enlarged right ventricle dimensions, and dilated pulmonary arteries.

Levosimendan is an inodilator drug considered a dependable therapeutic choice for managing both acute and advanced heart failure [60]. It is also being studied recently as a therapeutic option for PH-HFpEF patients [61].

Levosimendan provides numerous advantages for patients with PH-HFpEF, including decreased left ventricular filling pressure, better endothelial function, improved right ventricular systolic performance, and optimized right ventricle-pulmonary artery coupling. These benefits stem from reduced pulmonary artery resistance, elastance, and enhanced right ventricular contractility [62]. The effects of this drug on PH-HFpEF patients were recently studied in the HELP (Levosimendan Improves Hemodynamics and Exercise Tolerance in PH-HFpEF) trial [63]. Following the run-in period, which required hemodynamic improvements after 24 h of levosimendan, the study randomized 37 patients with HFpEF-PH (PAMP ≥ 35 mmHg) to receive either levosimendan or a placebo. While the study did not achieve its primary goal of reducing exercise PAWP at 25 watts after six weeks, levosimendan did result in a significant reduction in PAWP at all stages of exercise (−3.9 ± 2.0 mm Hg; *p* = 0.047) when compared to placebo, as well as decreases in central venous pressure. Furthermore, there was a statistically significant difference of 29.3 m in the six-minute walk distance between the two groups (*p* = 0.033). Although levosimendan is recognized for its potential inotropic and vasodilator effects, it has been hypothesized that hemodynamic benefits in patients with HFpEF appear to derive from a reduction in circulating blood volume, indicating that venodilation may be levosimendan’s primary mechanism of action in PH-HFpEF [64]. Although these early findings are encouraging, a phase 3 clinical trial is required to validate the effectiveness of levosimendan for treating patients with PH-HFpEF. Additionally, creating an oral version of levosimendan, which is currently being researched, is crucial for its broader application among these patients.

## 6. GLP-1 Agonists: A New Player Comes?

GLP1 is an incretin hormone produced by intestinal enteroendocrine cells (L cells) responding to nutrients within the intestinal lumen. Its primary function is to enhance insulin secretion from β cells in the pancreas while simultaneously reducing glucagon release from α cells. This dual action helps prevent postprandial blood glucose variations [65]. However, GLP1 also exhibits several additional effects, including boosting brown fat thermogenesis in adipose tissue and suppressing the hypothalamic hunger center, leading to substantial weight loss, especially in obese and insulin-resistant individuals [66].

In recent years, new inhibitors targeting enzymes that degrade natural GLP-1 have become available, positively influencing metabolic dysfunction. Research has demonstrated that GLP-1 agonists improve metabolic parameters—lowering hemoglobin A1c and significantly aiding in weight loss—while decreasing the rates of major adverse cardiovascular events, mainly by reducing ischemic episodes, in patients with type 2 diabetes mellitus [67]. The GLP-1 receptor is found in various human tissues, including the myocardium and the vascular smooth muscle cells of the middle layer of the pulmonary arteries. Thus, due to their diverse effects (see Table 2), GLP-1 agonists may play a significant role in patients with PH-HFpEF. Notably, their ability to reduce endothelial dysfunction—by enhancing nitric oxide production and lowering endothelin-1 synthesis—and induce vasodilation in pulmonary vessels may be crucial for the purported beneficial outcomes of GLP-1 agonists in managing PH-HFpEF.

In vitro studies indicate that GLP1 agonists lessen inflammation and enhance nitric oxide production, thereby inhibiting endothelial-to-mesenchymal transition. In this complex biological process, endothelial cells transform into a mesenchymal phenotype with typical morphology and functions commonly associated with PH [68]. Furthermore, in a bleomycin-induced model of idiopathic pulmonary fibrosis, liraglutide reduced the mRNA levels of collagen, hydroxyproline, and essential enzymes for collagen synthesis, leading to decreased fibrosis [69].

Moreover, GLP1 agonists positively influence ventricular performance. In the MAGNetic resonance Assessment of VICTOza efficacy in the Regression of cardiovascular dysfunction In type 2 diAbetes mellitus (MAGNA VICTORIA) trial, 49 patients with diabetes mellitus and HFpEF were randomized to receive either liraglutide (n = 23) or a placebo (n = 26). The trial demonstrated that liraglutide led to a significant reduction in the E/Ea value [−1.8 (−3.0 to −0.6); *p* < 0.005] as assessed via cardiac MRI [70].

In another study involving 60 patients with type 2 diabetes, participants were randomized to either liraglutide (n = 30) or metformin (n = 30). In this study, patients receiving liraglutide exhibited a notable improvement in carotid-femoral pulse wave velocity (11.8 ± 2.5 vs. 10.3 ± 3.3 m/s; *p* = 0.01), left ventricular global longitudinal strain (−15.4 ± 3 vs. −16.6 ± 2.7; *p* = 0.04), and a significant reduction in NT-proBNP levels (432 (154–2921) vs. 282 (80–2302); *p* = 0.03) after six months of treatment. These findings indicate that a six-month treatment with liraglutide enhances arterial stiffness and left ventricular myocardial strain and lowers NT-proBNP levels [71].

In conclusion, while preliminary, the evidence indicates that GLP1 agonists positively influence cardiac hemodynamics, mainly by facilitating left ventricular unloading. This effect, along with the reduction in insulin resistance and weight loss, likely leads to beneficial outcomes for PH-HFpEF patients, especially those with obesity as a comorbidity.

## 7. Conclusions

Recently, there has been considerable advancement in understanding the pathophysiology of PH-HFpEF, leading to the preliminary clinical application of ARNI and SGLT2i in this area. Notably, SGLT2i, approved for HF patients regardless of left ventricular ejection fraction, has demonstrated significant reductions in PASP and notable improvements in associated symptoms. Although sacubitril/valsartan is not yet licensed in many Western countries for the treatment of HFpEF, it has shown efficacy in patients presenting with symptoms of PH-HFpEF, especially in those already treated with SGLT2i and with a left ventricular ejection fraction of around 50%. Finerenone is a novel non-steroidal mineralocorticoid receptor antagonist with remarkable cardioprotective and renoprotective properties and has been shown in experimental studies to reduce PASP and pulmonary artery wall muscularization and thickness. Levosimendan would seem to represent a valid therapeutic option for PH-HFpEF patients; subsequent confirmation in phase 3 randomized clinical trials and the market entry of an oral formulation will represent a significant step forward for treating PH-HFpEF. Lastly, Preclinical evidence points to a possible role for GLP-1 agonists in treating patients with PH-HFpEF, particularly those with obesity.

## Figures and Tables

**Table 1:** Classical pathophysiological factors involved in PH-HFpEF.

Vascular	Hemodynamics Effects
Arterial wall stiffening	Increase of afterload
Abnormal vasorelaxation (impaired endothelial function)	Increase of afterload
**Cardiac**	
Cardiomyocytes hypertrophy	Increase of end-diastolic volume pressure relationship
Myocardial fibrosis	Increase of end-diastolic volume pressure relationship
Impaired coronary reserve	Increase of end-diastolic volume pressure relationship
**Pulmonary**	
Right ventricular failure	Increase left atrial pressure/pulmonary vascular resistance
Capillary remodeling	Increase left atrial pressure/pulmonary vascular resistance
Lung capillary stress failure	Increase left atrial pressure/pulmonary vascular resistance

**Table 2:** Pleiotropic effects of GLP1.

**Brian**
Appetite suppression
Nerve protection
Reduction of neuroinflammation
**Gastrointestinal tract**
Reduction of gastric emptying
**Heart**
Reduction of blood pressure
Reduction of serum lipid levels
Reduction of endothelial dysfunction
**Pulmonary arteries**
Vasodilatation
**Liver**
Reduction of glucose production
**Pancreas**
Increase in insulin secretion
Reduction of glucagon secretion
Induction of βcells’ islet proliferation
